# Something Worth Fighting for

**Published:** 1883-07

**Authors:** 


A State Board of Examiners, the greatest need of the Medical Profession in the State of New York—A "Mixed Board" the only efficient one attainable under present conditions.

### SOMETHING WORTH FIGHTING FOR.

We ask the especial attention of our readers to the report of the proceedings of the Erie County Medical Society contained in this issue. The feature of the meeting was the action upon the resolutions relative to the appointment of a "State Board of

Examiners." The question which divided the society was, as to the endorsement of the principle of "Mixed Boards."

There was no division of sentiment as to the necessity of a State Board of Examiners, and there was a perfect unanimity in the vote upon the second resolution, providing for a mixed Board like that of Illinois. But the frank endorsement of the principle upon which that Board is founded, and without which it would be not only futile, but impossible to have an effective Board at all, was warmly contested, and carried by a small majority.

It is not easy to satisfactorily explain the division which took place over the third resolution in view of the entire unanimity with which the second resolution was carried; but we think that the real feeling and sentiment of the society upon the question is evinced by the unanimous passage of the second resolution.

The JOURNAL is in favor of each and all of the resolutions as passed, and we are so, because we realize that the society has taken the initial step in a great work for a great cause, and it is expedient and necessary that there should be made and announced a platform plainly setting forth the principles to be maintained, and which can not be tortured by its enemies into a mere manifestation of sectarianism. We rejoice that the Erie County Society has had the honor of taking the initiative in this matter. We propose to support them in it with all our power, and we believe that when the matter is fairly put before the profession and the public, the movement will receive so earnest and hearty a support as will ensure the passage of a law creating such a Board, and endowing it with full power to raise the profession of medicine above the degrading influence of cheap and purchased diplomas. We hope to see the time when medical men shall win fromall the acknowledgement that theirs is a learned, an efficient, a capable and a liberal profession; but we believe that such a recognition will be neither deserved nor won until there shall come a complete separation of the unfortunate, unwise and debasing co-partnership of the educating and the licensing powers. That this movement will receive the strongest opposition from the medical schools is undoubtedly true, and it would be idle to ignore the power which will be exerted by the well-organized and determined opposition of corporate bodies backed with an abundance of money and a willingness to use it freely to accomplish their ends. It is not, perhaps, safe to count upon it, but let us hope that at least some of the better colleges will place the interests of the profession above the desire for "filthy lucre" and throw their influence upon the side of right. It is but justice to the Buffalo Medical College to say that every member of the faculty present at the meeting voted for a State Examining Board. We hope that our college will join with the Erie County Society in leading the van in the coming battle for the elevation of the profession.

